# Intelligent pressure-controlled retrograde intrarenal surgery versus microchannel percutaneous nephrolithotomy to treat 2–3 cm renal calculi

**DOI:** 10.1007/s00240-025-01799-w

**Published:** 2025-06-28

**Authors:** Zhongsheng Yang, Qiliang Zhai, Junjing Wu, Leming Song, Yongming Huang, Ting Sun

**Affiliations:** 1 Department of Urology, The Affiliated Ganzhou Hospital, Jiangxi Medical College, Nanchang University, Ganzhou, China; 2 Department of Urology, The First Affiliated Hospital, Jiangxi Medical College, Nanchang University, Nanchang, China

**Keywords:** Intelligent controlled-pressure, Retrograde intrarenal surgery, Microchannel percutaneous nephrolithotomy, Renal calculi

## Abstract

We performed a comparative analysis of intelligent pressure-controlled ureteroscopic lithotripsy (IRIRS) and intelligent pressure-controlled microchannel percutaneous nephrolithotomy (IMPCNL) to treat 2–3 cm renal calculi. Patients (*n* = 140) with 2–3 cm renal calculi were randomly divided into the IRIRS and IMPCNL groups (*n* = 70/group). Surgical time, length of hospital stays, stone clearance rate, decrease in hemoglobin level, postoperative pain score, and incidence of complications were compared between groups. The IRIRS group had significantly longer operative durations than the IMPCNL group (average: 58.3 ± 7.4 vs. 52.5 ± 6.8 min). Stone clearance rates at 4 weeks postoperatively were 90.0% and 92.8% in the IRIRS and IMPCNL groups, respectively (*P* > 0.05). The IRIRS group had shorter hospital stays than the IMPCNL group (1.9 ± 0.3 vs. 3.2 ± 0.4 days). Intraoperative hemoglobin levels decreased more in the IMPCNL (1.03 ± 0.12 g/dL) than in the IRIRS (0.25 ± 0.06 g/dL, *P* < 0.001) group. Two and one cases in the IRIRS and IMPCNL group, respectively, experienced postoperative fever. The overall complication incidence did not significantly differ (12.9% IRIRS and 15.7% IMPCNL; *P* > 0.05). IRIRS and IMPCNL are safe, effective interventions for 2–3 cm renal calculi. Given its minimally invasive nature and positive operative safety outcomes, IRIRS has promising future applications.

## Introduction

Urinary tract stones are highly prevalent in China, with an incidence rate of 5.5–11.6% in the southern region. It is among the most common diseases treated in urology departments [1]. Retrograde intrarenal surgery (RIRS) and percutaneous nephrolithotomy (PCNL) are the primary methods for treating kidney stones. RIRS—a minimally invasive procedure performed through natural body cavities—offers several advantages, including minimal damage, reduced bleeding, and quick recovery. However, it is a significant disadvantage associated with low stone fragmentation efficiency. Conversely, PCNL has the advantages of high stone fragmentation efficiency and fragmented stone removal; however, it is associated with risks of significant bleeding and long hospitalization stays [2–4]. Currently, PCNL is generally recommended for kidney stones larger than 2 cm, while RIRS is commonly used for smaller stones. However, with technological advancements, RIRS is gradually becoming feasible for kidney stones larger than 2 cm [5–7].

**Fig. 1:**
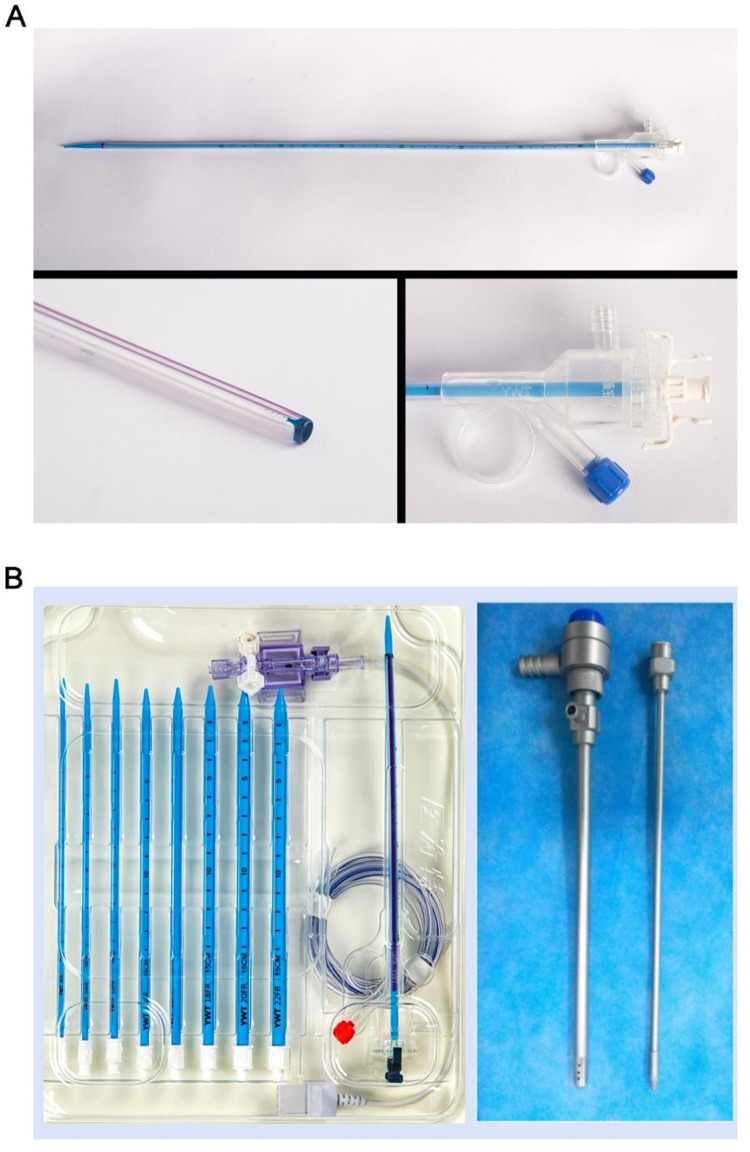
The pressure-measuring suction sheath. (**A**) Manometric ureteral suction sheath. (**B**) Manometric percutaneous renal suction sheath with metal and disposable sheaths

Renal pelvic pressure, a critical factor for intracorporeal lithotripsy, may be high due to improper irrigation and inadequate drainage. Elevated pressure in the renal pelvis can lead to varying degrees of kidney damage, fluid reflux and extravasation, spread of infection, urinary sepsis, and septic shock [8, 9]. To address the challenge of elevated renal pelvic pressure and improve surgical effectiveness, we developed pressure-measuring suction sheaths – both ureteral and percutaneous nephrostomy sheaths (Fig. 1A and B).

These innovative devices enable easy renal pelvic pressure monitoring and facilitate the pressure data transmission to an irrigation and suction system equipped with pressure feedback control (medical irrigation and suction pressure control platform). By employing pressure feedback control to regulate suction strength, intrapelvic pressure can be automatically and precisely controlled and maintained within a safe range. This approach mitigates complications associated with high renal pelvic pressure, including urinary sepsis, fluid extravasation, and kidney damage. The application of a novel intelligent pressure control system in intracorporeal lithotripsy suction stone removal surgery further enables intelligent monitoring and control of renal pelvic pressure, ensuring the desired irrigation flow rate and enhancing surgical efficiency through effective stone suction.

**Fig. 2:**
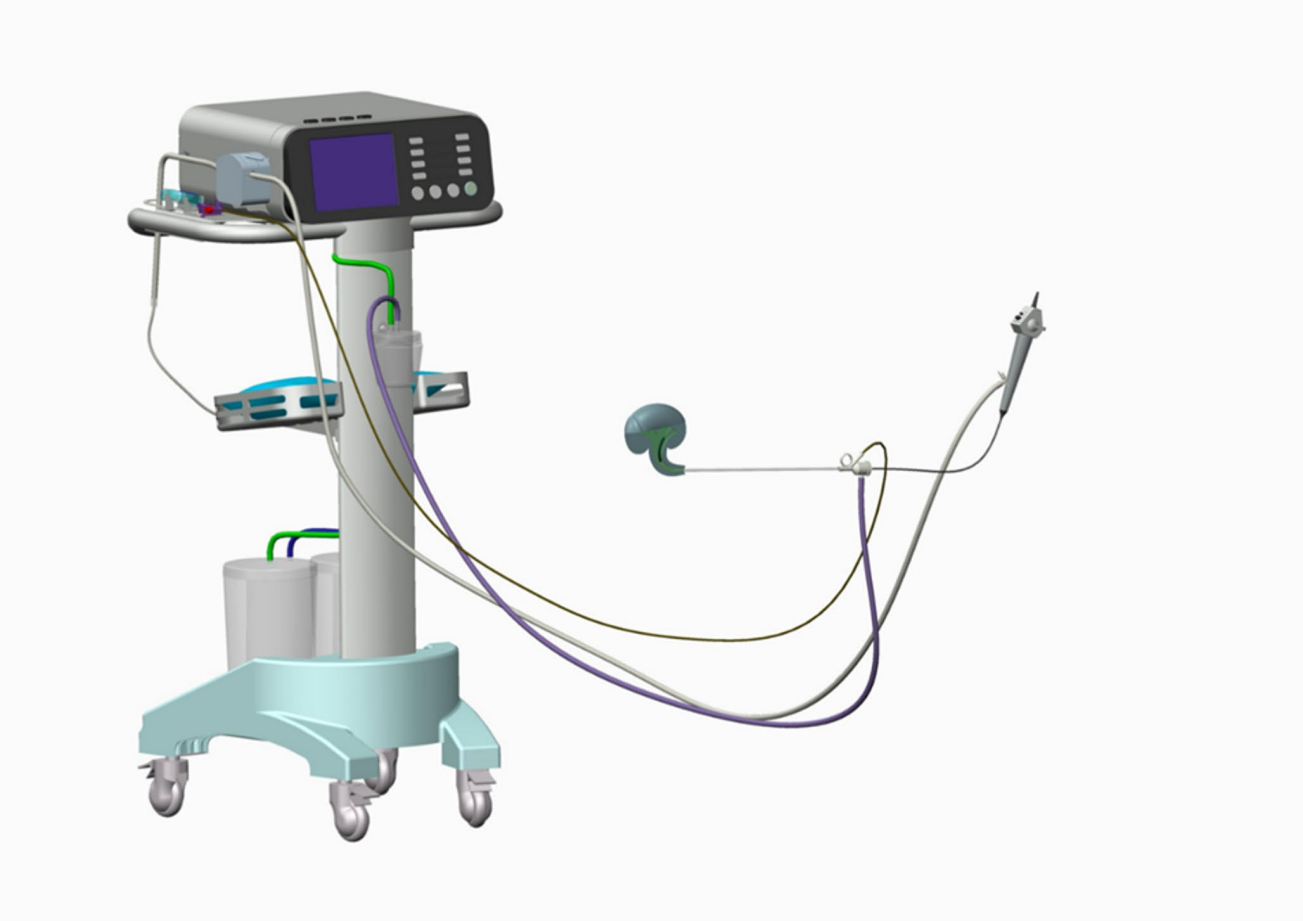
The schematic of the IRIRS

Significant improvements in surgical efficiency and safety have been achieved by applying intelligent pressure-controlled intracavitary lithotripsy. Intelligent pressure-controlled ureteroscopic lithotripsy (IRIRS)(Figure 2) has shown promising clinical efficacy in the treatment of kidney stones larger than 2 cm [10]. Similarly, intelligent controlled-pressure IMPCNL has demonstrated favorable outcomes in kidney stone treatment [11]. However, selecting the appropriate surgical method for 2–3 cm renal calculi remains challenging. Therefore, we performed a comparative study to evaluate the efficacy and safety of IRIRS and IMPCNL for treating 2–3 cm renal calculi.

## Methods

### Study participants

This study was approved by the Ethics Committee of the Affiliated Ganzhou Hospital of Nanchang University. The Project Number is ky2014014. We enrolled 140 patients with 2–3 cm renal calculi (Urological CT and abdominal X-ray)who were admitted to our hospital for inpatient treatment between January 2019 and January 2021. The inclusion criteria were as follows: (1) solitary pelvic or calyx stones up to 2–3 cm in diameter; (2) aged 18–65 years; (3) male or female. The exclusion criteria were as follows: (1) patients with bleeding disorders, severe cardiopulmonary insufficiency, severe spinal deformity, morbid obesity, acute infection, urinary tract anomalies, and pregnancy; (2) patients with renal tuberculosis or renal tumors; (3) patients with a history of ureteral stenosis.

The patients were divided into two treatment groups (IRIRS and IMPCNL) of 70 patients each, according to the random number table method.

## Surgery

Patients in the IRIRS group were placed under general anesthesia and positioned in the lateral decubitus position on their healthy side. The ureter was examined using a rigid ureteroscope (KARL STORZ 8–9.5 Fr, Tuttlingen, Germany), and a guidewire(with a diameter of 0.028 inches) was placed retrogradely into the renal pelvis. A ureteroscopic suction sheath (12/14 Fr)capable of pressure measurement was inserted until it reached the ureteropelvic junction. The pressure measurement channel of the ureteral sheath was connected to a hydraulic sensor, which was, in turn, connected to the irrigation and suction platform for pressure measuring. The suction channel of the ureteral sheath was connected to a suction tube, which led to a stone collection bottle on the medical irrigation and suction platforms. A peristaltic irrigation tube was installed. Once all the connections were established, the mode was switched to automatic irrigation and suction. Normal saline solution was injected to eliminate air from the sensor and pressure measurement channel of the ureteral sheath, thus effectively zeroing the intracavity pressure. The desired irrigation flow rate (50–120 mL/min), intracavity pressure control value (−2 to −9 mmHg), and intracavity pressure alarm value (30 mmHg) were configured. The literature supports 30 mmHg as the “safe threshold” for intrapelvic pressure. Subsequently, IRIRS was performed. Insert the single-use digital flexible ureteroscope (REDPINE^®^ 8Fr Model RP-U-C-12, Guangzhou, China) through the ureteral access sheath, then a holmium laser(Lumenis^®^ VersaPulse^®^ PowerSuite 100 W, Yokneam, Israel) fiber with a diameter of either 200–365 μm (1.0–1.6 J × 30 Hz) was inserted to fragment the stone. The stone fragments were suctioned out through the gap between the sheath and irrigation channel using water flow. In the case of larger stone particles, the flexible ureteroscope was withdrawn to aid suction. Stone fragmentation and suction were repeated alternately. During suctioning, the irrigation flow rate was increased to 100–120 mL/min. Gentle tapping of the kidney area was performed to facilitate stone suctioning. For stones located far from the ureteral sheath, the direction of irrigation and suction flow were carefully considered. In such cases, the stone was initially suctioned closer to the sheath (e.g., near the renal pelvis) before removal. After renal pelvis and calyces examination, standard F5 ureteral stent and urinary catheter placement were performed postoperatively.

**Fig. 3:**
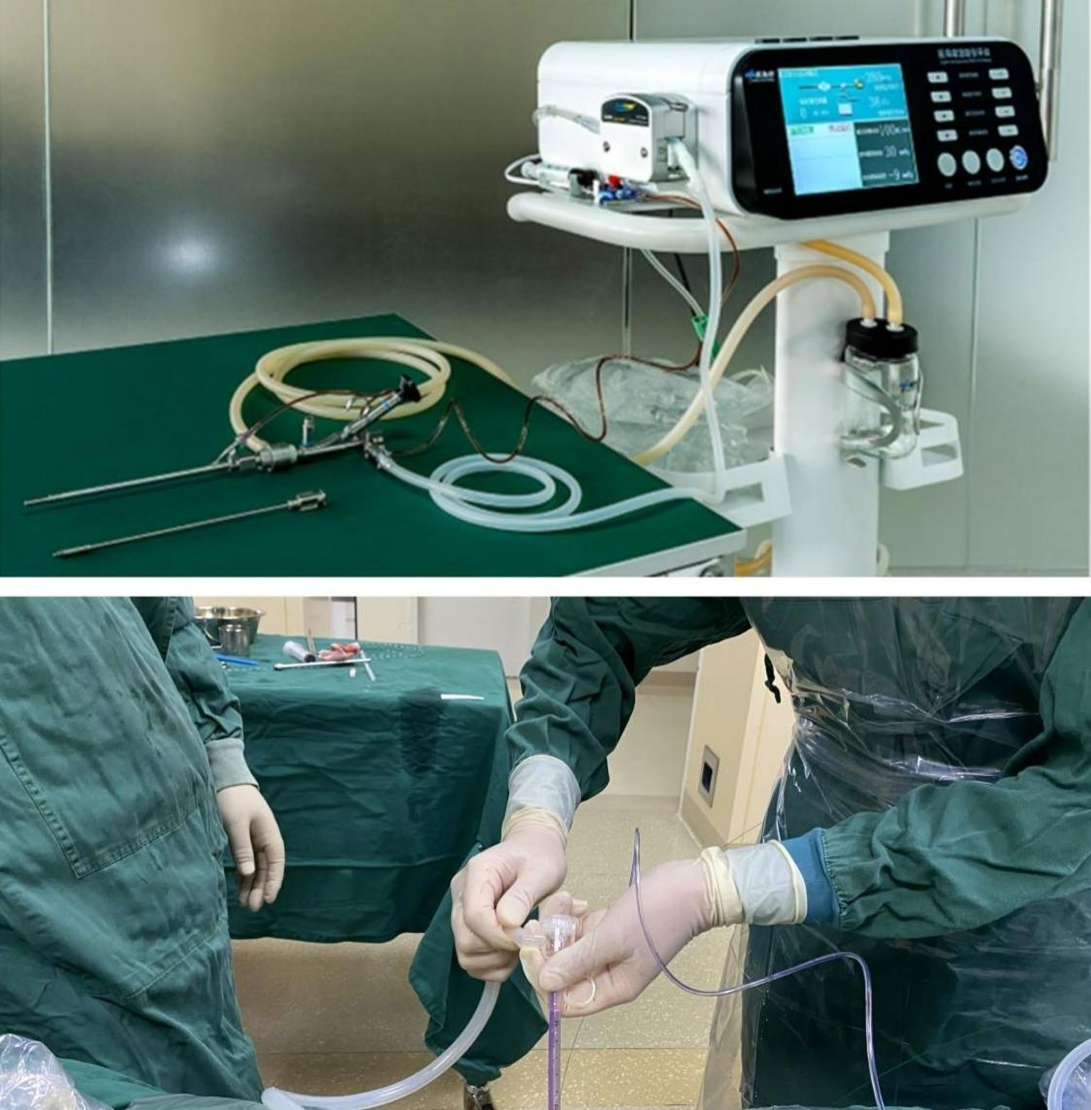
The main equipment and connections of IMPCNL

Patients in the IMPCNL group were positioned for stone retrieval under general or combined spinal anesthesia. A ureteral catheter was inserted through the urethra, advanced into the renal pelvis, and secured. The patient was then placed in a prone position without abdominal elevation. Guided by ultrasonography, a percutaneous renal access tract was established via skin puncture. A microchannel suction sheath (inner diameter 16Fr, outer diameter 18Fr) capable of pressure measurement was inserted. The sheath pressure monitoring channel was connected to a pressure sensor, further linked to a medical perfusion suction platform via a pressure-measuring port. The sheath suction channel was then connected to a suction tube linked to a stone collection bottle on the medical perfusion suction platform (Fig. 3). The interface of the nephroscopic perfusion channel was then connected to an infusion tube on the same platform. Subsequently, the medical perfusion suction platform was switched to the automatic infusion suction mode. The desired infusion flow rate (200–500 mL/min), intracavity pressure control value (–2 to –5), and intracavity pressure alarm value (30 mmHg) were set on the platform control panel. After setting the platform parameters, the intracavity pressure was calibrated to zero. A high-power holmium laser with a diameter of 550 μm (2.5 J × 30 Hz) with a Wolf nephroscope (8Fr, Knittlingen, Germany) was used to perform stone fragmentation and dusting. The stone fragments were suctioned using negative pressure applied through the sheath. Due to the negative pressure suction, the renal pelvis or calyx stones were not easily displaced, facilitating fragmentation and preventing stone particle dispersion. Renal pelvic pressure could be adjusted based on fragmentation requirements, ensuring a clear view and improving continuous stone fragmentation and clearance efficiency. Abdominal elevation was avoided to facilitate kidney movement and stone fragmentation. Further, as stones in large-angle calyces can be pushed into the renal pelvis using a sheath before fragmentation, we ensured no residual stones. If the neck of the smaller calyx was narrow during the examination, a sheath was used to dilate it appropriately before performing stone fragmentation and clearance. After ensuring that no residual stones were present, a routine F6 ureteral stent was placed, with or without nephrostomy tube placement, depending on the intraoperative situation.

The surgical time, bleeding volume (measured based on the decrease in hemoglobin level), need for blood transfusion and need for any intervention surgery were recorded. Documented complications included infections (fever exceeding 38.5 °C and presence of sepsis), visual analog scale (VAS) pain scores, ureteral injuries, and intraoperative damage to the surrounding organs. After 4 weeks, follow-up examinations were performed using kidney-ureter-bladder radiography and computed tomography (CT) to record the stone clearance rate. Other information, including hospitalization duration, was also recorded.

## Statistical analyses

Statistical analysis was performed using SPSS (version 26.0). Continuous, normally distributed data are expressed as mean ± standard deviation. Student’s t-test was used to compare groups. Categorical data are presented as n (%), and the chi-square test was used for comparisons. Statistical significance was set at *P* < 0.05.

## Results

Overall, 140 patients were enrolled, with 70 in each treatment group. The IRIRS group comprised 34 males and 36 females (average age, 50 ± 5.85 years; maximum stone diameter, 2.43 ± 0.36 cm; stone CT value, 976.8 ± 105.7 HU). The IMPCNL group comprised 37 males and 33 females (average age, 48 ± 5.15 years; maximum stone diameter, 2.47 ± 0.32 cm; stone CT value, 990.4 ± 114.7 HU). The groups did not significantly differ in age, sex, stone size, or CT values.

In the IRIRS group, placing the sheath during the initial surgery was difficult in one patient because of a ureteral stricture. Following the indwelling of a ureteral stent for 2 weeks, a second-stage flexible ureteroscopy was successfully performed. In the IMPCNL group, all surgeries were considered successful – a percutaneous renal access channel was established without causing serious complications such as ureteral tears or organ damage. The duration of IRIRS (58.3 ± 7.4 min) was longer than that of IMPCNL (52.5 ± 6.8 min), although non-significantly (*P* = 0.12). The stone clearance rate was slightly lower in the IRIRS group (63/70, 90.0%) than in the IMPCNL group (65/70, 92.8%), although non-significantly (*P* = 0.546). Intraoperative bleeding (Haemoglobin decline)was significantly lower in the IRIRS (0.25 ± 0.06 g/dL) than in the IMPCNL (1.03 ± 0.12 g/dL; *P* < 0.001) group. Postoperatively, in the IMPCNL group, one patient required a blood transfusion, while three had significant hematuria but recovered with conservative treatment. In the IRIRS and the IMPCNL groups, six and five patients, respectively, experienced postoperative fever (body temperature > 38.5 °C; *P* = 0.65). All patients were treated with antibiotics and recovered. There were no cases of sepsis and no statistically significant differences between the groups. Postoperative pain scores were significantly lower in the IRIRS (2.6 ± 0.2) than in the IMPCNL (4.4 ± 0.5; *P* < 0.05) group. Two patients experienced damage to the ureteral mucosa in the IRIRS group. One patient experienced urinary extravasation in the IMPCNL group. Nine and eleven postoperative complications occurred in the IRIRS and IMPCNL groups, respectively (*P* = 0.629). The length of hospital stay was significantly shorter in the IRIRS (1.9 ± 0.3 days) than in the IMPCNL (3.2 ± 0.4 days, *P* = 0.014) group (Table 1).

**Table 1:** Comparison of efficacy and complications between the two groups

Projects	IRIRS group	IMPCNL group	*P*-value
Operative time (min)	58.3 ± 7.4	52.5 ± 6.8	0.128
Haemoglobin decline (g/L)	2.5 ± 0.6	10.3 ± 1.2	0.001
Postoperative fever, n (%)	6 (8.6%)	5 (7.1%)	0.791
Postoperative VAS pain	2.6 ± 0.2	4.4 ± 0.5	0.025
Overall complications, n, %	9 (12.9%)	11 (15.7%)	0.629
Stone clearance rate at 4 weeks post-surgery, n (%)	63 (90.0%)	65 (92.8%)	0.546
Hospitalization time (days)	1.9 ± 0.3	3.2 ± 0.4	0.014

IMPCNL, intelligent controlled-pressure microchannel percutaneous nephrolithotomy; IRIRS, Intelligent controlled-pressure retrograde intrarenal surgery; VAS, visual analogue scale

All patients were followed up for 6–24 months (average follow-up, 12 months). Five and three patients in the IRIRS and IMPCNL groups, respectively, had recurrent stones requiring repeat surgery. Postoperative follow-up revealed that one patient in each group had narrowing at the junction of the renal pelvis and ureter accompanied by hydronephrosis. These patients underwent Laparoscopic Pyeloplasty.

## Discussion

As RIRS is increasingly performed for ureteral stone fragmentation, flexible ureteral endoscopes, and related devices are being continuously developed. With increased surgical experience, RIRS efficiency for stone fragmentation has continuously improved while complications have decreased. Consequently, the indications for RIRS have been expanding [12, 13]. Typically, the irrigation flow during stone fragmentation is maintained between 50 and 100 mL/min and is increased to 50–120 mL/min during stone clearance. Recently, we have developed an intelligent pressure-controlled ureteral flexible endoscope stone retrieval technique that significantly enhances pressure monitoring, control, and stone retrieval [10]. This has facilitated a substantial increase in irrigation flow during the procedure, circumventing limitations such as poor visibility, reduced stone fragmentation efficiency, and thermal injury risk. The significant flow rate increase notably enhanced visibility during stone fragmentation, consequently improving process speed and efficiency. For renal pelvic stones with mild hydronephrosis, upper/mid-calyceal stones, and lower-calyceal stones with a large renal pelvic angle, patients were positioned on their healthy side in the lateral decubitus position to ensure ureteral orifice alignment with the lowest point of the renal pelvis and calyces, thus facilitating stone suction removal by water flow. As such, complete stone pulverization may not always be necessary, and most small stone fragments can be suctioned, thereby reducing stone fragmentation time. In our study, the average operative time for RIRS was less than 1 h, demonstrating a significant improvement in surgical efficiency and a relatively high stone clearance rate. As most of the stones are removed during this surgery, the postoperative ureteral stone repeat occurrence and reoperation requirement in recurrent cases are relatively rare.

With advances in intelligent controlled-pressure intracavitary lithotripsy technology, this technology has been increasingly applied to microchannel PCNL to improve the safety and surgical efficiency of PCNL, yielding promising results in clinical practice. Herein, the intelligent controlled-pressure approach in microchannel PCNL demonstrated notable clinical efficacy, low complication rates, high stone clearance rates, and short operative times. Overall, it is feasible to increase the perfusion rate to enhance the stone field visibility during stone fragmentation and clearance. Furthermore, suction significantly increases the fluid circulation speed within the renal pelvis, thereby aiding in the removal of stone fragments and expediting the efficiency of stone clearance.

Renal calculi with 2–3 cm have a relatively small stone burden; therefore, the microchannel approach was appropriate, as it minimized trauma. The application of the new instrument ensured that the intrarenal pressure could be maintained within a predetermined safety range across various perfusion rates.

We explored the efficacy and safety of IRIRS and IMPCNL for 2–3 cm renal calculi treatment. Our results indicate that the stone clearance rate of IRIRS was slightly lower than that of IMPCNL for treating 2–3 cm renal calculi. The IMPCNL had a higher stone fragmentation efficiency than the IRIRS, particularly for stones with high CT values [14]. Furthermore, post-PCNL, fragmented stones can be immediately suctioned out. In contrast, IRIRS involves pulverization before using water flow suction or stone retrieval baskets to remove large fragments. Consequently, intelligently controlled pressure techniques significantly enhanced the stone clearance rate, enabling the removal of most fragmented stones [15]. However, in cases involving lower-calyceal stones with narrow infundibulopelvic angles or significant hydronephrosis, stone suctioning could pose challenges, necessitating natural clearance. Our study suggests no significant difference in stone clearance rates between IRIRS and IMPCNL 4 weeks postoperatively, indicating a significant improvement in stone fragmentation efficiency of IRIRS compared to previous techniques. This improvement was probably due to the use of novel devices, the ability to suction the majority of stones, and the capacity of flexible endoscopes to facilitate a comprehensive inspection of various calyces and fragmented stones. Our findings also showed that the operative time and postoperative infection rate were slightly higher in the IRIRS than in the IMPCNL group. However, in terms of operation time, IMPCNL outperformed IRIRS because of its relatively high stone fragmentation and retrieval efficiency. Intelligent controlled-pressure techniques effectively curtailed intrapelvic pressure, thus reducing operation duration and concurrently decreasing the incidence of postoperative complications, such as infections. No cases of severe sepsis were reported in this study. The elevated incidence of infection (postoperative fever) in IRIRS may be attributed to challenges in ureteral sheath positioning and occasional transient high local pressure, although percutaneous renal access channels are wide and short. Intelligently controlled pressure techniques created a mildly negative pressure state in the renal pelvis, although these differences were not statistically significant.

Regarding intraoperative bleeding, length of hospital stays, and postoperative VAS pain scores, the IRIRS performed significantly better than the IMPCNL. IRIRS is performed through natural body cavities, whereas IMPCNL requires percutaneous channel establishment through the vascular-rich renal cortex. Trauma induced by IMPCNL was significantly more severe than that caused by IRIRS, which led to increased blood loss, a higher likelihood of requiring blood transfusions and, consequently, a slower recovery. For patients presenting with lower-pole stones or hard renal stones with high CT values, as well as those with moderate-to-severe hydronephrosis, percutaneous nephrolithotomy with intelligent pressure control can deliver superior surgical outcomes and reduce the likelihood of stone recurrence.

Owing to the advantages of minimal trauma, rapid recovery, limited bleeding, and short hospital stays compared to those of IMPCNL, an increasing number of patients and physicians are opting for IRIRS as a treatment choice. However, follow-up observations in our study revealed a high probability of recurrence and the need for reoperation in the patients after ureteroscopic treatment. Therefore, patients should consider personalized and precise treatment plans that suit their individual needs. For patients with lower-pole stones or hard kidney stones with high CT values and those with moderate-to-severe hydronephrosis, PCNL with intelligent pressure control can achieve improved surgical outcomes with a low likelihood of stone recurrence.

There is still room for improvement in intelligent pressure control equipment. The pressure-sensing element is located at the front of the pressure-measuring sheath. However, in flexible ureteroscopy surgery, the pressure-measuring sheath is often positioned in the upper segment of the ureter rather than within the renal pelvis. Consequently, the measured pressure reflects the pressure in the space where the pressure-measuring sheath is located, potentially leading to underestimated pressures. The generation of stone debris and blood clots during surgery can partially obstruct the pressure-measuring sheath [16]. Hydraulic sensors are often integrated into the main pressure control unit, with a relatively long conduction distance from the pressure-measuring hole at the front of the sheath to the sensor. This may result in energy loss over distance and a delayed response time, leading to pressure measurement deviations [17]. These three factors may contribute to imprecise pressure measurements during the surgical procedure.

This study investigated intelligent pressure-controlled ureteroscopy and PCNL in treating larger (2–3 cm) kidney stones. The aim was to explore a more minimally invasive, efficient, safe, and cost-effective treatment method. The goal is to promote widespread application, benefiting a large number of patients, ensuring patient safety, and reducing medical costs. This approach aims to advance the field of minimally invasive stone removal surgery toward intelligent control of intracavitary pressure. Further research should explore ways to improve the existing pressure control equipment and the accuracy and real-time performance of the current pressure measurement methods to refine efficiency and safety. Our study suggests that for patients with kidney stones of 2–3 cm, IRIRS may be a preferable option due to its minimally invasive nature, lower complication rates, and quicker recovery.

This study has several limitations. The small sample size and single-center design may limit the generalizability of the findings. Additionally, the follow-up period may be insufficient to fully assess long-term stone recurrence and complications. While key surgical outcomes were evaluated, comprehensive indicators such as long-term renal function and patient quality of life were not thoroughly assessed. Future research should include larger multi-center prospective randomized controlled trials with extended follow-ups and refined evaluation criteria to validate these findings and optimize treatment strategies.

## Data Availability

No datasets were generated or analysed during the current study.
